# Exploring Factors, Comorbidities, Quality of Life (DLQI), and Depression (PHQ‐9) in Rosacea Patients: A Comprehensive Analysis

**DOI:** 10.1111/jocd.70337

**Published:** 2025-07-31

**Authors:** Namrata Kunwar, Dan Jian, He Yan Hui, Bingxue Bai

**Affiliations:** ^1^ Dermatology and Venereology Department The Second Affiliated Hospital of Harbin Medical University Harbin Heilongjiang Province People's Republic of China

**Keywords:** acne rosacea, comorbidities, DLQI (Dermatology Life Quality Index), PHQ‐9 (Patient Health Questionnaire‐9), QoL (quality of life)

## Abstract

**Background:**

Rosacea is a chronic inflammatory skin condition primarily affecting the face, which greatly influences patients' quality of life and mental well‐being.

**Aim:**

This research aimed to examine environmental and dietary factors, systemic comorbidities, and their effects on the quality of life and psychological impact of rosacea in Chinese patients.

**Methods:**

The study was an observational case–control design involving 200 individuals with rosacea and 200 healthy participants. Participants completed a questionnaire addressing skin color, specific habits, dietary and environmental factors, comorbidities, and assessments of quality of life and depression levels using the Dermatology Life Quality Index (DLQI) and the Patient Health Questionnaire‐9 (PHQ‐9), respectively.

**Results:**

The chi‐square test was employed to compare the distribution of general characteristics. Univariate analysis was conducted using Pearson's chi‐square test. DLQI is a continuous scale analyzed via independent *t*‐test and PHQ‐9 is an ordinal scale analyzed via Mann–Whitney *U* test. Spearman's rank correlation coefficient was used for nonparametric correlation between two ranked variables. A *p* value < 0.05 was considered statistically significant. Chi‐square results with OR and 95% CI indicated significant associations between rosacea and factors such as female gender, age, smoking, alcohol consumption, environmental influences (sun exposure, use of cosmetics/hair products, exercise, exposure to hot weather, hot baths), dietary factors (dairy products, hot coffee consumption), and comorbidities (diabetes mellitus). No significant links were found with skin colors, dietary factors (fatty food, hot and spicy food, hot tea, sweet food), or other comorbidities (hypertension, high cholesterol, *Helicobacter pylori* , cancer, Crohn's/ulcerative colitis, dry eye syndrome, chronic kidney disease, and migraine). The rosacea group showed notably higher DLQI scores with moderate effect (9.0%), large effect (40.5%), and extremely large effect (45.5%), along with elevated PHQ‐9 scores for depression compared to controls, highlighting a significant impact on their quality of life and mental health. Spearman's rank correlation coefficient demonstrated a significantly positive correlation between DLQI and PHQ‐9 scores among participants (rho = 0.423, *p* < 0.001), indicating that greater quality of life impairment is linked to increased depression levels.

**Conclusions:**

These results underscore the complex nature of rosacea and its significant psychosocial burden. Addressing modifiable risk factors (e.g., smoking cessation, avoid alcohol, hot coffee and dairy products intake, sun protection, cessation of cosmetic products) and incorporating (DLQI/PHQ‐9 screening) mental health screening and psychological support into treatment could enhance patient outcomes. Further research is necessary to clarify causal mechanisms and improve management strategies, which will aid in preventing and improving rosacea conditions and treatment approaches in the future.

## 1 Introduction

Rosacea is a chronic inflammatory condition affecting the facial skin, presenting a variety of skin manifestations such as erythema, telangiectasia, papules, pustules, and thickened skin changes [1]. This disorder is characterized by increased inflammatory cytokines and infiltration of immune cells, with its pathophysiology involving a complex interaction of genetic factors, neurovascular irregularities, abnormal immune responses, and microbial influences like Demodex mites and *Staphylococcus epidermidis* [2, 3, 4, 5, 6, 7]. Clinically, rosacea is divided into four subtypes: (i) papulopustular rosacea (PPR), featuring papules and pustules with ongoing redness; (ii) erythematotelangiectatic rosacea (ETR), marked by flushing, persistent redness, or telangiectasia; (iii) phymatous rosacea (PhR), which includes skin thickening, irregular nodules, and enlargement; and (iv) ocular rosacea, which involves eye‐related symptoms [5]. Rosacea is more commonly seen in individuals with Fitzpatrick skin types I–II and those of Celtic or Northern European ancestry [3]. Its global prevalence is not well studied, especially in large populations like those in China and Australia. Reported prevalence rates vary significantly, from 5.0% in Russia to 12.3% in Germany, with an estimated 13 million people affected in the United States [4].

Recent findings emphasize the multifaceted nature of rosacea, where environmental factors—such as temperature changes, UV exposure, stress, smoking, alcohol, skin microbial imbalances, and spicy foods—worsen cutaneous vasodilation, inflammatory processes, and neovascularization, leading to redness and facial telangiectasias [8, 9]. Beyond skin manifestations, rosacea is increasingly associated with systemic comorbidities. Observational studies link it to cardiovascular diseases, metabolic disorders (like diabetes and dyslipidemia), gastrointestinal conditions (such as inflammatory bowel disease), and neurological disorders including migraines, Parkinson's disease, and depression [10, 11, 12, 13, 14]. There is also a suggested link between autoimmune diseases and certain cancers [15], highlighting the broader systemic impact of this condition.

The psychosocial impact of rosacea is significant, often affecting quality of life (QoL) and mental health. Visible facial lesions and flushing can result in social stigma, anxiety, and depressive symptoms [13, 16, 17]. Tools like the Dermatology Life Quality Index (DLQI) and the Patient Health Questionnaire‐9 (PHQ‐9) are essential for measuring these effects. The DLQI, widely used in dermatology, evaluates disease‐specific QoL impairments [18, 19, 20], while the PHQ‐9, an instrument aligned with the Diagnostic and Statistical Manual of Mental Disorders (DSM‐IV) with 88% sensitivity and specificity, provides a reliable measure of depression severity [21, 22]. Despite these tools, the psychological effects of rosacea are often overlooked, particularly in Chinese populations where cultural views on skin health may increase distress.

Regional studies are urgently needed in China, where the prevalence of rosacea and its psychosocial effects are not well documented. This study aimed to (1) identify demographic, clinical, and environmental factors associated with rosacea in China; (2) evaluate its impact on quality of life using the DLQI; and (3) assess the prevalence and severity of depression using the PHQ‐9. By clarifying regional risk factors and psychosocial burdens, this research seeks to inform targeted interventions to improve disease management and mental health outcomes for those affected.

## 2 Materials and Methods

### 2.1 Study Design and Subjects

This research is an Observational Case and Control Study. The research protocol has been reviewed and granted an exemption from formal ethics approval by an ethics committee of the Second Affiliated Hospital of Harbin Medical University, as the study involved minimal risk, anonymous questionnaires with implied consent. Prior to completing the questionnaires, all participants provided an explanation and verbal informed consent obtained. The study included 200 rosacea patients diagnosed at the dermatology outpatient department of the Second Affiliated Hospital of Harbin Medical University from May 2018 to May 2020. Additionally, 200 healthy controls were selected from the general population (the family members accompanying the patients, Students and families of hospital employees). The inclusion criteria were: (1) patients diagnosed with rosacea exhibiting clinical symptoms of the condition; and (2) ability to comprehend the questionnaire content. The exclusion criteria were: (1) exclusion of foreign patients, meaning only individuals of Chinese origin were included; and (2) patients with facial eczema, facial contact dermatitis, solar dermatitis, or other facial inflammatory skin diseases were not included. Healthy controls were individuals without any skin diseases.

### 2.2 Data Collection

All survey participants provided verbal consent. Board‐certified dermatologists assessed skin condition, lesion location, and rosacea symptoms in the outpatient setting. A trained resident doctor administered a standardized questionnaire during outpatient visits. The study aimed to identify skin color, specific habits, dietary and environmental factors, comorbidities, and assessments of quality of life and depression levels associated with rosacea in patients seeking treatment, so various factors potentially linked to rosacea were included. Comorbidities were verified via medication and medical records. The questionnaire was in a pen‐and‐paper format, translated into Chinese to ensure participants could easily understand the questions.

### 2.3 Questionnaires

The questionnaires consisted of three sections: (1) General information such as age, gender, skin color, and specific habits (e.g., smoking and alcohol consumption). Dietary habits of the Chinese population, including the intake of hot and spicy foods, fried foods, dairy products, hot tea, and hot coffee, were also considered. Factors that might trigger the disease, such as sun exposure, exercise, hot weather, hot baths, and the use of cosmetics or hair products, were recorded. Systemic comorbidities were self‐reported and confirmed through medication and medical records. These comorbidities included hypertension, diabetes mellitus, high cholesterol, *Helicobacter pylori* infection, cancer, Crohn's disease or ulcerative colitis, dry eye syndrome, chronic kidney disease, and migraine. (2) DLQI—The 10 questions were divided into six categories. The total DLQI score is classified into five grades. A score of (0–1) corresponds to Grade 1, indicating no impact on the patient's life. A score of (2–5) indicates a small impact, (6–10) a moderate impact, (11–20) a very large impact, and (21–30) an extremely large impact on the patient's life. (3) PHQ‐9 Questionnaire—This section included 9 questions. The total PHQ‐9 score is divided into five levels of depression. A score of (0–4) indicates no depression, (5–9) minimal depression, (10–14) minor depression, and (15–19) moderate depression. A score of (20–27) severe depression (Data S1).

### 2.4 Statistical Analysis

We used the chi‐square test to compare the distributions of general characteristics between the rosacea and control groups. Categorical variables were analyzed with Pearson's chi‐square test. DLQI is a continuous variable analyzed via independent *t*‐test and PHQ‐9 is an ordinal variable analyzed via Mann–Whitney *U* test. A value of *p* < 0.05 was considered to be statistically significant. Spearman's rank correlation coefficient is used for nonparametric measure of correlation between two ranked variables. Data were analyzed using the IBM SPSS Statistics, Version 30.

## 3 Results

### 3.1 General Characteristics of Study Participants

Our research involved 400 individuals, comprising 200 rosacea patients and 200 healthy controls. Among them, 300 were women, with 141 being cases (70.5%) and 159 as healthy controls (79.5%), while 100 were men, including 59 cases (29.5%) and 41 healthy controls (20.5%). The age distribution showed a statistical significance between the rosacea and control groups. Participants were categorized into age groups: under 19, 20–29, 30–39, 40–49, and over 50 years. No significant link was found between skin color and rosacea. Participants' skin tones were classified as pale, light brown, fair white, brown, dark white, and dark brown. Of these, 162 had light brown skin (76 cases [38%] and 86 healthy controls [43%]), 127 had fair white skin (68 cases [34%] and 59 healthy controls [29.5%]), 24 had pale skin (9 cases [4.5%] and 15 healthy controls [7.5%]), 57 had brown skin (31 cases [15.5%] and 26 healthy controls [13%]), 13 had dark white skin (8 cases [4%] and 5 healthy controls [2.5%]), and 17 had dark brown skin (8 cases [4%] and 9 healthy controls [4.5%]). Participant characteristics are detailed in Table 1.

**TABLE 1:** Comparison of general characteristics between the rosacea and control groups (*n* = 400).

Characteristic	Control group, *n* (%)	Rosacea group, *n* (%)	*ꭓ* ^2^	*p*
Sex			4.320	0.038
Female	159 (79.5)	141 (70.5)		
Male	41 (20.5)	59 (29.5)		
Age			68.834	0.040
< 19	2 (1)	1 (0.5)		
20–29	33 (16.5)	30 (15)		
30–39	43 (21.5)	50 (25)		
40–49	66 (33)	52 (26)		
> 50	56 (28)	67 (33.5)		
Skin color			3.945	0.557
Pale	15 (7.5)	9 (4.5)		
Light brown	86 (43)	76 (38)		
Fair brown	59 (29.5)	68 (34)		
Brown	26 (13)	31 (15.5)		
Dark white	5 (2.5)	8 (4)		
Dark brown	9 (4.5)	8 (4)		

### 3.2 Associated Factors Between Rosacea and Control Data

The rosacea group had a higher number of smokers compared to the control group (20% vs. 7%, *p* < 0.001). Similarly, alcohol consumption was more prevalent in the rosacea group than in the control group (22% vs. 5.5%, *p* < 0.001). Sun exposure or sunburn was significantly more associated with rosacea than with the control group (47.5% vs. 0.0%, *p* ≤ 0.001). The use of cosmetics and hair products was notably higher among those with rosacea compared to the controls (29.5% vs. 0.0%, *p* ≤ 0.001). Our findings indicate that rosacea is more prevalent in hot weather, with a significantly greater effect observed in the rosacea group than in the control group (29.5% vs. 0.0%, *p* ≤ 0.001). Daily exercise (5.0% vs. 0.0%, *p* ≤ 0.001) and taking hot baths (12.6% vs. 0.0%, *p* ≤ 0.001) were also significantly associated with rosacea compared to the control group, as shown in Table 2.

**TABLE 2:** Associated factors between rosacea and control data.

Characteristic	Control group, *n* (%)	Rosacea group, *n* (%)	*ꭓ* ^2^	OR	95% CI	*p*
Smoking status			14.472	3.321	1.744–6.326	< 0.001
Yes	14 (7)	40 (20)				
No	186	160				
Alcohol usage			22.957	4.846	2.421–9.699	< 0.001
Yes	11 (5.5)	44 (22)				
No	189	156				
Sunburn/exposure to sunlight			124.590	0.525	0.460–0.599	< 0.001
Yes	0 (0.0)	95 (47.5)				
No	200	105				
Cosmetics/hair products usage			69.208	0.705	0.645–0.771	< 0.001
Yes	0 (0.0)	59 (29.5)				
No	200	141				
Exercise			10.256	0.950	0.920–0.981	< 0.001
Yes	0 (0.0)	10 (5.0)				
No	200	190				
Hot weather			69.208	0.705	0.645–0.771	< 0.001
Yes	0 (0.0)	59 (29.5)				
No	200	141				
Hot bath			26.805	0.874	0.830–0.922	< 0.001
Yes	0 (0.0)	25 (12.6)				
No	200	174				

### 3.3 Comparison on the Food Intake Between the Rosacea and Control Groups

According to chi‐square test results, the consumption of dairy products (milk, yogurt) was significantly higher among controls compared to those with rosacea (44.5% vs. 29%, *p* = 0.001). Similarly, hot coffee consumption was significantly greater in the control group than in the rosacea group (14.5% vs. 8%, *p* = 0.040). There was no significant difference in the intake of fatty foods, hot and spicy foods, sweet foods, and hot tea between the two groups. Only 28.5% of rosacea patients and 30.5% of the control group consumed fatty foods. Both the groups had a 52.5% intake of hot and spicy foods. Sweet food consumption was 43.5% among rosacea patients and 38.5% among controls. Hot tea consumption was observed in 10% of rosacea patients and 13% of controls. The intake of fatty foods, sweet foods, hot and spicy foods, and hot tea did not show an association with rosacea in our study, as detailed in Table 3.

**TABLE 3:** Comparison of the food intake between the rosacea and control groups.

Food items	Control group, *n* (%)	Rosacea group, *n* (%)	*ꭓ* ^2^	OR	95% CI	*p*
Fried food			0.192	0.908	0.591–1.396	0.661
Yes	61 (30.5)	57 (28.5)				
No	139	143				
Dairy products (milk, yogurt)			10.336	0.509	0.337–0.770	0.001
Yes	89 (44.5)	58 (29)				
No	111	142				
Hot and spicy food			0.000	1.000	0.675–1.481	1.000
Yes	105 (52.5)	105 (52.5)				
No	95	95				
Sweet food			1.033	1.230	0.825–1.833	0.309
Yes	77 (38.5)	87 (43.5)				
No	123	113				
Hot tea			0.884	0.744	0.400–1.381	0.347
Yes	26 (13)	20 (10)				
No	174	180				
Hot coffee			4.232	0.513	0.269–0.977	0.040
Yes	29 (14.5)	16 (8)				
No	171	184				

### 3.4 Comparison of Systemic Comorbidities Between the Rosacea and Control Groups

There was only a significant association of diabetes mellitus between the rosacea and control groups. Five percent of rosacea patients with diabetes mellitus versus 1.5% of controls, *p* = 0.048. The occurrence of systemic comorbidities like hypertension, high cholesterol, *H. pylori* , cancer, Crohn's/ulcerative colitis, dry eye syndrome, and migraine/headache did not differ significantly between the two groups according to chi‐square test results. Twelve percent of rosacea patients present with hypertension while 9.5% were observed in the control group. High cholesterol was present in 9.5% of cases and 5.5% of controls in this study. Similarly, *H. pylori* was present in 4.0% of the rosacea group and 2.5% of the control group. Cancer was observed in only 2.5% of rosacea and 1.0% of controls. One percent of rosacea patients present with Crohn's/ulcerative colitis while 1.5% were observed in the control group. Likewise, dry eye syndrome was present in 14.5% of rosacea patients and 12.5% of controls in our study. 2.5% of patients presented with chronic kidney disease and only 0.5% of controls presented with chronic kidney disease. 26% of rosacea patients presented with migraine while 25% of controls presented with migraine. In our study, hypertension, high cholesterol, *H. pylori* , cancer, Crohn's/ulcerative colitis, dry eye syndrome, chronic kidney disease, and migraine/headache failed to show a positive association with rosacea (Table 4).

**TABLE 4:** Comparison of systemic comorbidities between the rosacea and control groups.

Comorbidities	Control group, *n* (%)	Rosacea group, *n* (%)	*ꭓ* ^2^	OR	95% CI	*p*
Hypertension			0.651	1.299	0.687–2.455	0.420
Yes	19 (9.5)	24 (12)				
No	181	176				
Diabetes mellitus			3.896	3.456	0.937–12.752	0.048
Yes	3 (1.5)	10 (5)				
No	197	190				
High cholesterol			2.306	1.804	0.835–3.896	0.129
Yes	11 (5.5)	19 (9.5)				
No	189	181				
*Helicobacter pylori*			0.716	1.625	0.522–5.056	0.398
Yes	5 (2.5)	8 (4.0)				
No	195	192				
Cancer			1.309	2.538	0.487–13.240	0.253
Yes	2 (1.0)	5 (2.5)				
No	198	195				
Crohn's/ulcerative colitis			0.203	0.663	0.110–4.013	0.653
Yes	3 (1.5)	2 (1.0)				
No	197	198				
Dry eye syndrome			0.343	1.187	0.668–2.110	0.558
Yes	25 (12.5)	29 (14.5)				
No	175	171				
Chronic kidney disease			2.707	5.103	0.591–44.073	0.100
Yes	1 (0.5)	5 (2.5)				
No	199	195				
Migraine/headache			0.053	1.054	0.672–1.653	0.819
Yes	50 (25)	52 (26)				
No	150	148				

### 3.5 Comparison of DLQI Score and PHQ‐9 Score Between the Rosacea and Control Groups

**FIGURE 1:**
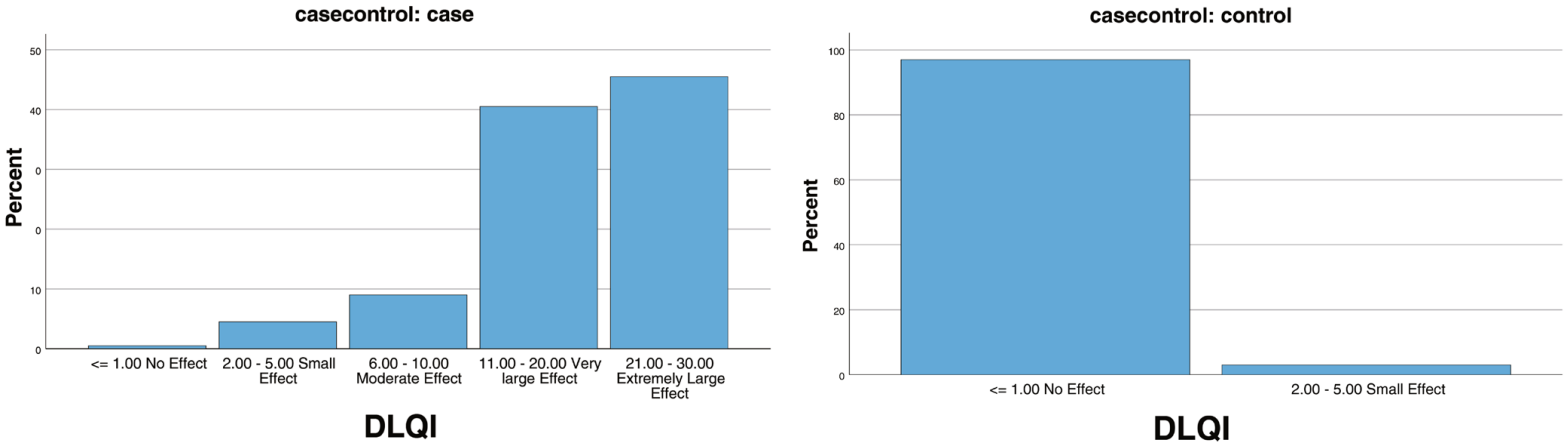
Distribution of DLQI score in the rosacea and control groups.

The rosacea group obtained significantly higher DLQI scores than the control group (Table 5). Total DLQI score showed significantly more patients with moderate effect (9.0%), large effect (40.5%), extremely large effect (45.5%) in the rosacea group compared with the control group with no effect (97.0%) and small effect (3.0%) (*p* < 0.001, Figure 1).

**TABLE 5:** DLQI score of the rosacea and control groups ($\bar{X}$ ± S).

	Rosacea	Control	*t*‐test	*p*
Symptoms and feelings				
Question 1	1.76 ± 0.922	0.03 ± 0.171	26.028	< 0.001
Question 2	1.53 ± 1.065	0.03 ± 0.171	19.662	< 0.001
Daily activities				
Question 3	1.90 ± 1.017	0.03 ± 0.171	25.633	< 0.001
Question 4	1.95 ± 0.998	0.03 ± 0.171	26.734	< 0.001
Leisure				
Question 5	1.85 ± 0.967	0.03 ± 0.171	26.131	< 0.001
Question 6	2.17 ± 0.919	0.02 ± 0.140	32.692	< 0.001
Work and school				
Question 7	1.77 ± 1.479	0.03 ± 0.171	16.525	< 0.001
Personal relationship				
Question 8	2.40 ± 0.750	0.03 ± 0.171	43.505	< 0.001
Question 9	1.22 ± 0.907	0.03 ± 0.171	18.151	< 0.001
Treatment				
Question 10	2.22 ± 0.857	0.02 ± 0.140	35.807	< 0.001
Total score	2.65 ± 1.727	1.50 ± 0.501	130.00	< 0.001

**FIGURE 2:**
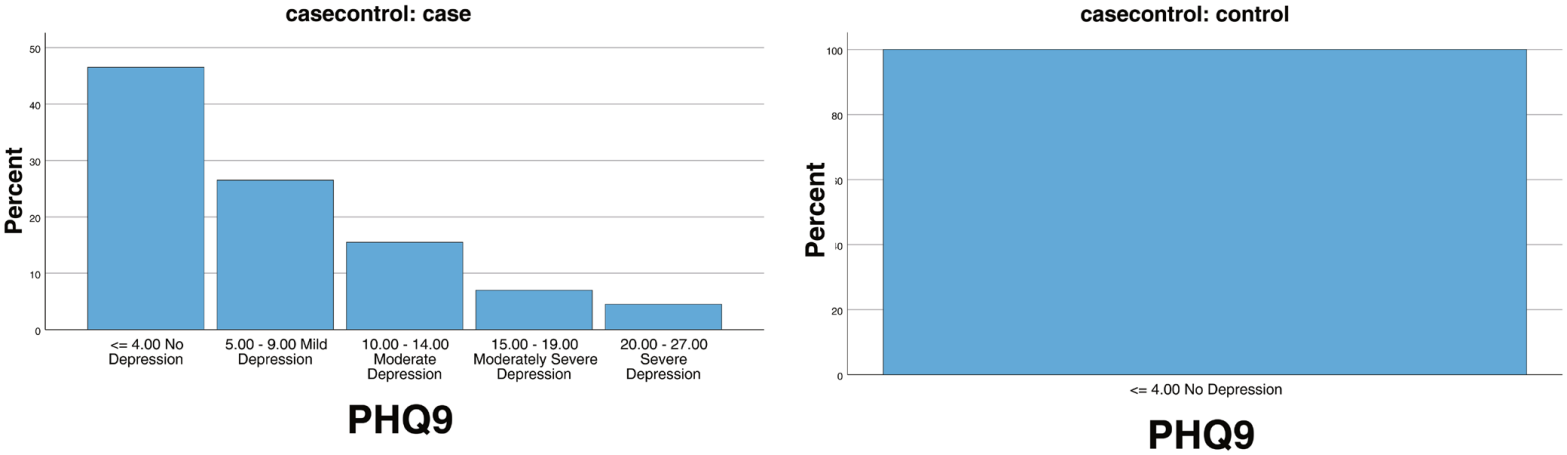
PHQ‐9 score. Depression level among rosacea (*N* = 200) and control (*N* = 200).

In PHQ‐9, rosacea patients had significantly higher scores of depression compared with the control group. In total PHQ‐9 score, no depression (46.5%), mild depression (26.5%), moderate depression (15.5%), moderately severe depression (7.0%), and severe depression (4.5%) reported in Rosacea patients compared with the control group, with 100% reporting No Depression (Table 6, Figure 2).

**TABLE 6:** PHQ‐9 score (*N* = 400).

PHQ‐9 score	*N*	Percent (%)
Case		
≤ 4.00 no depression	93	46.5
5.00–9.00 mild depression	53	26.5
10.00–14.00 moderate depression	31	15.5
15.00–19.00 moderately severe depression	14	7.0
20.00–27.00 severe depression	9	4.5

PHQ‐9 score	Case, mean ± SD	Control, mean ± SD	Mann–Whitney, *U* test	*p*
Total PHQ‐9 score	1.97 ± 1.145	1.00 ± 0.000	9300.000	< 0.001

The relationship between DLQI scores and PHQ‐9 scores was examined using Spearman's rank correlation coefficient. The analysis revealed a significant positive correlation between DLQI scores and PHQ‐9 scores among the participants (rho = 0.423, *N* = 400, *p* < 0.001). This suggests that higher DLQI scores are associated with higher PHQ‐9 scores (Table 7).

**TABLE 7:** Spearman's Rho correlation between PHQ‐9 and DLQI.

Spearman's rho correlations	PHQ‐9	DLQI
PHQ‐9		
Correlation coefficient	1.000	0.423 (< 0.001)
Sig.(2‐tailed)		< 0.001
DLQI		
Correlation coefficient	0.423 (< 0.001)	1.000
Sig.(2‐tailed)	< 0.001	

## 4 Discussion

Environmental elements, especially dietary factors, are believed to be associated with the progression of rosacea. Previous studies have investigated the pathophysiology of rosacea, systemic comorbidities, and associated environmental risk factors of rosacea [23]. Our single‐center case–control study analyzed the associations between environmental elements, including dietary factors, systemic comorbidities, and psychosocial impacts (including quality of life and depression) in rosacea patients. We will now contextualize our results within existing research and highlight new insights.

Our study identified significant associations between rosacea and smoking (OR = 3.321; 95% CI: 1.75–6.33, *p* < 0.001) and alcohol consumption (OR = 4.846; 95% CI: 2.42–9.67, *p* < 0.001). These outcomes are consistent with a US study by Li et al., which noted higher rates of smoking among rosacea patients [24] The angiogenic effects of nicotine may explain this connection, particularly in ETR [25] While the role of alcohol in rosacea remains controversial, our findings suggest it may worsen disease progression. This contrasts with French survey data where only 5.6% of participants associated flare‐ups with alcohol [26]. Further population‐specific studies are warranted to clarify this discrepancy.

Our research revealed a notable positive correlation between the consumption of dairy products and the incidence of rosacea (*χ* ^2^ = 10.336, *p* = 0.001). This finding contradicts earlier studies that suggested dairy might play a protective role in reducing rosacea severity by enhancing intestinal epithelial barrier function, modulating the gut microbiome, and consequently decreasing inflammation. The discrepancy in results could be attributed to variations in the types and quantities of dairy consumed across different Chinese populations, lactose intolerance rates in Chinese adults, and fermentation differences [10, 27]. Interestingly, our research found no link between spicy food consumption and the development of rosacea, despite its common association with triggering flushing symptoms. This suggests that spicy foods may only exacerbate symptoms rather than cause the condition. Additionally, our findings showed no correlation between rosacea and the intake of fatty or sweet foods, challenging theories that connect insulin resistance to the disease's origin. In contrast to studies conducted on Chinese populations, where caffeine consumption is typically lower, our research identified hot coffee as a potential risk factor for rosacea [27, 28]. On the other hand, our data did not support a significant connection between tea consumption and rosacea, despite existing theories proposing that excessive tea intake might contribute to ETR via neurovascular dysfunction through sympathetic activation [6, 29]. This suggests that other mechanisms may be more influential in our study population.

There are various triggers found to exacerbate rosacea, which include sun exposure, high temperature, exercise, hot bath, cosmetics, or hair products [28]. In line with extensive survey findings [30], sun exposure demonstrated a significant correlation with rosacea, likely attributed to UV‐induced oxidative stress and the activation of the KLK5‐cathelicidin cascade. The observed seasonal fluctuations, particularly exacerbations during summer, aligned with data from the National Rosacea Society [31]. However, conflicting reports underscore the necessity for long‐term climate studies. The use of cosmetic and hair products also proved relevant, consistent with our clinical observations [32]. Studies showed that heavy exercise and hot baths/showers lead to flushing. Interestingly, our study showed significant correlations between heavy exercise and hot baths with rosacea.

In terms of coexisting conditions, a significant correlation (*p* < 0.05) was found between rosacea and diabetes mellitus, lending support to previous theories of metabolic dysfunction [10]. However, the study did not reveal any connections with hypertension, *H. pylori* , dyslipidemia, chronic kidney disease, or dry eye syndrome, which contradicts some existing research [33]. It is worth noting that our investigation failed to establish any link between rosacea and inflammatory bowel disease (IBD) or cancer, which differs from findings in Southern Chinese populations. The study performed in southern China revealed increased risk of neurological and breast cancer with decreased risk of hematological cancer in rosacea. However, our study did not investigate any association between cancer and rosacea [16, 34]. These discrepancies might be attributed to variations in genetic makeup or environmental factors.

In our study, the DLQI score for rosacea patients was significantly higher than that of healthy controls. 45.5% of rosacea patients reported severe quality of life impairment. As compared to controls, rosacea patients had more subjective symptoms, experiencing embarrassment or low self‐esteem, which would further affect their daily activities and social relationships. Our findings are in line with what previous studies have found [17, 19, 35, 36, 37].

Furthermore, our study showed that PHQ‐9 scores were higher in the rosacea patients compared to controls. As per my knowledge, this is the first study showing the positive correlation between DLQI score and PHQ‐9 score. Because of this, the doctors must pay attention to patients with higher DLQI scores to provide them with psychological counseling as early as possible.

Rosacea had greatly affected the psychological well‐being of Chinese patients and significantly impacted their overall quality of life. Physicians should pay attention to the psychological needs of patients with rosacea in addition to treating their physical symptoms.

While this study provides valuable insights, several limitations should be acknowledged. First, the single‐center design and relatively small sample size. Second, self‐reported data on dietary habits and environmental exposures could introduce recall bias. Third, the cross‐sectional design precludes establishing causal relationships between rosacea and identified factors. Fourth, the homogeneous Chinese population may not reflect global diversity. Fifth, lack of rosacea subtype stratification. Sixth, potential Chinese cultural stigma influencing DLQI & PHQ‐9 score. Seventh, seasonal variation in trigger exposure (e.g., sun in summer).

## 5 Conclusion

In conclusion, our study suggests significant associations between rosacea and female gender, age, smoking, alcohol consumption, environmental triggers (sun exposure, cosmetics/hair products usage, hot weather), dietary factors (dairy products (milk, yogurt), hot coffee consumption) and diabetes mellitus. However, no significant associations were observed with skin colors, dietary factors (fatty food, hot and spicy food, hot tea, sweet food) and other comorbidities (hypertension, high cholesterol, *H. pylori* , cancer, Crohn's/ulcerative colitis, dry eye syndrome, chronic kidney disease and migraine). Rosacea patients had significantly higher DLQI and PHQ‐9 scores as compared to controls, indicating a substantial impact on their quality of life and mental health. The positive correlation between DLQI and PHQ‐9 scores suggested that higher impairment in quality of life is associated with increased depression levels. This supports integrating routine DLQI/PHQ‐9 screening into rosacea management to identify at‐risk patients requiring multidisciplinary care. Since the etiology of rosacea is still unclear, our study contributes to identifying the factors associated with rosacea, which will further help to prevent as well as improve rosacea conditions and treatment strategies in the near future.

These findings highlight the multifactorial nature of rosacea and its profound psychosocial burden. Addressing modifiable risk factors (e.g., smoking cessation, avoid alcohol, hot coffee and dairy products intake, sun protection, cessation of cosmetic products), integrating mental health screening and psychological support into treatment may improve patient outcomes. Further research with longitudinal, multicenter studies is needed to elucidate causal mechanisms and refine management strategies. It will further help to prevent as well as improve rosacea conditions and treatment strategies in the near future.

## Ethics Statement

The research protocol has been reviewed and granted an exemption from formal ethics approval by an ethics committee of Second Affiliated Hospital of Harbin Medical University as the study involved minimal risk, anonymous questionnaires with implied consent. Prior to completing the questionnaires, all participants provided explanation and verbal informed consent obtained.

## Conflicts of Interest

The authors declare no conflicts of interest.

## Supporting information


Data S1.

## Data Availability

The data that support the findings of this study are available from the corresponding author upon reasonable request.
