# Supplementary material for: Exploring Factors, Comorbidities, Quality of Life (DLQI), and Depression (PHQ‐9) in Rosacea Patients: A Comprehensive Analysis
Source: J Cosmet Dermatol. 2025 Jul 31;24(8):e70337. doi: 10.1111/jocd.70337 (PMC12312079; doi:10.1111/jocd.70337)
Supplement: Supplementary file 1 — Data S1. [file JOCD-24-e70337-s001.docx]

**1. Questionnaire**

**Q.1** Age: ___

**Q.2** Gender:

Male Female

**Q.3** In your opinion, what causes your Acne Rosacea (optional)? Choose the ones you feel might cause your rosacea.

**Q.3a** Sunburn or prolonged exposure to sunlight.

**Q.3b** Cosmetics or hair products

**Q.3C** Exercise

**Q.3d** Hot weather

**Q.3e** Hot baths

**Q.4** What kind of foods and drink you prefer to take? Tick the ones you often eat and drink.

**Q.4a** Fried foods

**Q.4b** Dairy products like milk, yoghurt

**Q.4c**  Hot and Spicy foods

**Q.4d** Sweet foods

**Q.4e** Hot Tea

**Q.4f** Hot Coffee

**Q.4g** Smoking

**Q.4h** Alcohol

**Q.5** Do you have any of the following disease: Tick the ones if you are suffering from any of the following.

**Q.5a**  Hypertension

**Q.5b**  Diabetes Mellitus

**Q.5c** High Cholesterol level

**Q.5d**  H. pylori

**Q.5e**  Cancer

**Q.5f** Ulcerative colitis / Crohn’s disease

**Q.5g** Dry eye syndrome

**Q.5h** Chronic kidney disease

**Q.5i** Migraine / Headache

**Q.6** Which one best describes the color of your normal skin? Choose one.

**1** - Pale

**2** - Light brown

**3** - Fair white

**4** - Brown

**5** - Dark white

**6** - Dark brown/white

**Dermatology Life Quality Index Questionnaire & Score (DLQI Score)**

*Q.6 Over the last week, have you felt the problem as mentioned. Tick the ones you have felt over the last week by using the following scale:*

*3 = very much; 2 = a lot; 1 = a little; 0 = not at all*

Q.6i Has your skin felt itchy, sore, painful or stinging? (Symptoms and feelings)

3 = very much; 2 = a lot; 1= a little; 0 = not at all

Q.6ii Have you felt embarrassed or self-conscious because of your skin? (symptoms and feelings)

3 = very much; 2 = a lot; 1 = a little; 0 = not at all

Q.6iii How much has your skin interfered with you going shopping, or looking after your home or garden? (daily activities)

3 = very much; 2 = a lot; 1 = a little; 0 = not at all

Q.6iv How much has your skin influenced the clothes you wear? Do you cover your face often? (daily activities)

3 = very much; 2 = a lot; 1 = a little; 0 = not at all

Q.6v How much has your skin affected any social or leisure activities? (LEISURE)

3= very much; 2 = a lot; 1 = a little; 0 = not at all

Q.6vi How much has your skin made it difficult for you to do any sport? ? (LEISURE)

3 = very much; 2 = a lot; 1 = a little; 0 = not at all

Q.6vii Has your skin prevented you from working or studying? (WORK & SCHOOL)

3 = Yes 0 = No

If “No” over the last week how much has your skin been a problem at work or studying?

2 = a lot; 1 = a little; 0 = not at all

Q.6 viii Has your skin problem caused problem with your partner or relatives or close friends? (PERSONAL RELATIONSHIPS)

3 = very much; 2 = a lot; 1 = a little; 0 = not at all

Q.6 ix How much of a problem has the treatment for your skin been, for example by making your home messy, or by taking up time? (TREATMENT)

3 = Very much; 2 = A lot; 1 = A little; 0 = Not at all

Q.6 x Over the last week, How much of a problem has the treatment for your skin been , for example by making your home messy , or by taking up time ?

3 = Very much ; 2 = A lot ; 1= A little ; 0 = Not at all

**Patient Health Questionnaire -9 ( PHQ-9 )**

Q.7 Over last 2 weeks, how often have you been bothered by following problems?

0 = Not at all; 1 = Several days; 2 = More than half the days; 3 = Nearly everyday

Q.7i Little interest or pleasure in doing things.

0 = Not at all; 1 = Several days; 2= More than half the days; 3 = Nearly everyday

Q.7ii Feeling down, depressed or hopeless.

0 = Not at all; 1 = Several days; 2= More than half the days; 3 = Nearly everyday

Q.7iii Trouble falling or staying asleep or sleeping too much

0 = Not at all; 1 = Several days; 2 = More than half the days; 3 = Nearly everyday

Q.7iv Feeling tired or having little energy.

0 = Not at all; 1 = Several days; 2 = More than half the days; 3 = Nearly everyday

Q.7v Poor appetite or overeating.

0 = Not at all; 1 = Several days; 2= More than half the days; 3 = Nearly everyday

Q.7vi Feeling bad about yourself.

0 = Not at all; 1 = Several days; 2 = More than half the days; 3 = Nearly everyday

Q.7vii Trouble concentrating on things, like reading newspaper or watching television.

0 = Not at all; 1= Several days; 2 = More than half the days; 3 = Nearly everyday

Q.7viii Moving or speaking so slowly that other people could have noticed.

0 = Not at all; 1 = Several days; 2= More than half the days; 3 = Nearly everyday

Q.7ix Thought of hurting yourself in some way or thought that you might feel you would better off dead.

0 = Not at all; 1 = Several days; 2 = More than half the days; 3 = Nearly everyday
